# Supplementary material for: Causal Relationship between Aging and Anorexia Nervosa: A White-Matter-Microstructure-Mediated Mendelian Randomization Analysis
Source: Biomedicines. 2024 Aug 16;12(8):1874. doi: 10.3390/biomedicines12081874 (PMC11351342; doi:10.3390/biomedicines12081874)
Supplement: Supplementary file 1 [file biomedicines-12-01874-s001.zip › Supplementary Figure/Figure S9/Leaveoneout plot of MR analysis for ubm-b-1940 to AN.pdf]

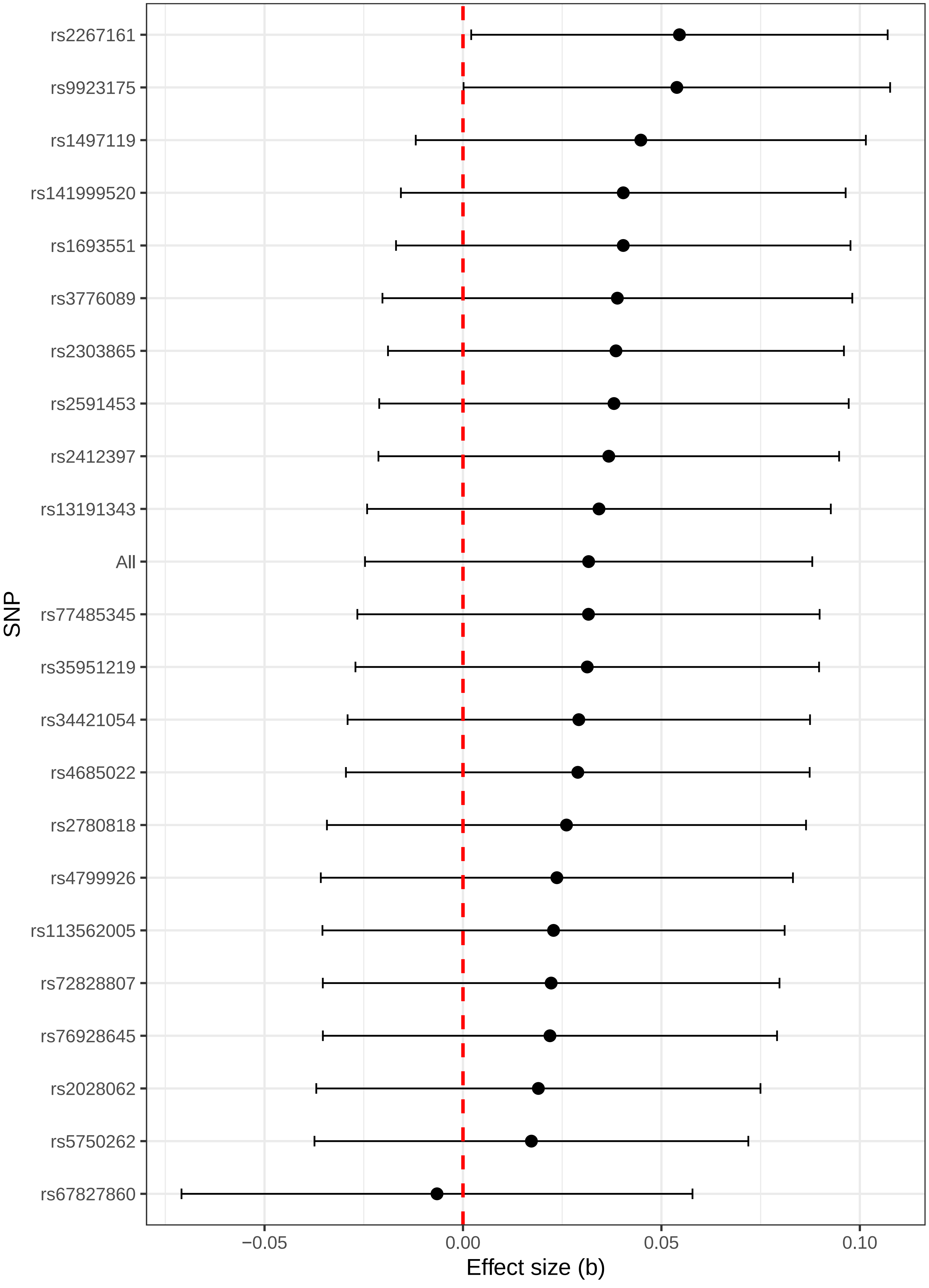

rs2267161
rs9923175
rs1497119
rs141999520
rs1693551
rs3776089
rs2303865
rs2591453
rs2412397
rs13191343
All
rs77485345
rs35951219
rs34421054
rs4685022
rs2780818
rs4799926
rs113562005
rs72828807
rs76928645
rs2028062
rs5750262
rs67827860
SNP
−0.05
0.00
0.05
0.10
Effect size (b)
